# Elevated Expression of Phospholipid Transfer Protein in Bone Marrow Derived Cells Causes Atherosclerosis

**DOI:** 10.1371/journal.pone.0002255

**Published:** 2008-05-28

**Authors:** Rien van Haperen, Hannelore Samyn, Matthijs Moerland, Teus van Gent, Marian Peeters, Frank Grosveld, Arie van Tol, Rini de Crom

**Affiliations:** 1 Department of Cell Biology and Genetics, Erasmus MC University Medical Center, Rotterdam, The Netherlands; 2 Department of Vascular Surgery, Erasmus MC University Medical Center, Rotterdam, The Netherlands; Duke University, United States of America

## Abstract

**Background:**

Phospholipid transfer protein (PLTP) is expressed by various cell types. In plasma, it is associated with high density lipoproteins (HDL). Elevated levels of PLTP in transgenic mice result in decreased HDL and increased atherosclerosis. PLTP is present in human atherosclerotic lesions, where it seems to be macrophage derived. The aim of the present study is to evaluate the atherogenic potential of macrophage derived PLTP.

**Methods and Findings:**

Here we show that macrophages from human PLTP transgenic mice secrete active PLTP. Subsequently, we performed bone marrow transplantations using either wild type mice (PLTPwt/wt), hemizygous PLTP transgenic mice (huPLTPtg/wt) or homozygous PLTP transgenic mice (huPLTPtg/tg) as donors and low density lipoprotein receptor deficient mice (LDLR−/−) as acceptors, in order to establish the role of PLTP expressed by bone marrow derived cells in diet-induced atherogenesis. Atherosclerosis was increased in the huPLTPtg/wt→LDLR−/− mice (2.3-fold) and even further in the huPLTPtg/tg→LDLR−/− mice (4.5-fold) compared with the control PLTPwt/wt→LDLR−/− mice (both P<0.001). Plasma PLTP activity levels and non-HDL cholesterol were increased and HDL cholesterol decreased compared with controls (all P<0.01). PLTP was present in atherosclerotic plaques in the mice as demonstrated by immunohistochemistry and appears to co-localize with macrophages. Isolated macrophages from PLTP transgenic mice do not show differences in cholesterol efflux or in cytokine production. Lipopolysaccharide activation of macrophages results in increased production of PLTP. This effect was strongly amplified in PLTP transgenic macrophages.

**Conclusions:**

We conclude that PLTP expression by bone marrow derived cells results in atherogenic effects on plasma lipids, increased PLTP activity, high local PLTP protein levels in the atherosclerotic lesions and increased atherosclerotic lesion size.

## Introduction

Phospholipid transfer protein (PLTP) is a plasma protein with the capacity to transfer phospholipids between liposomes and lipoproteins in vitro [1]–[4]. In addition, PLTP can also transfer vitamin E [5] and is active as a conversion factor of high density lipoproteins (HDL) [6], [7]. HDL are generally considered anti-atherogenic lipoproteins by virtue of their role in cholesterol excretion (reverse cholesterol transport) [8], although other anti-atherosclerotic properties of HDL have been described as well [9], [10]. Therefore, it was suggested that PLTP has a role in the development of atherosclerosis, based on its relation with HDL function [11], [12]. In vivo, phospholipids become available for PLTP-mediated transfer during lipolysis of triglyceride-rich lipoproteins, mostly chylomicrons and very low density lipoproteins (VLDL), by the enzyme lipoprotein lipase [12]. The phospholipids are transferred from triglyceride-rich lipoproteins to HDL. PLTP is able to bind to and facilitate the transfer of several types of phospholipids, including phosphatidylcholine, phosphatidylethanolamine, and phosphatidylinositol, as well as sphingomyeline [13]. In plasma, PLTP is mostly bound to HDL [11].

In genetically modified mouse models the relation between the activity levels of PLTP in plasma, HDL levels, HDL subclass distribution and the development of atherosclerosis was studied in more detail. We found that a 2.5-fold increased plasma PLTP activity in transgenic mice resulted in a 30% decrease in plasma HDL-cholesterol levels compared with wild type animals [14]. This represents total HDL, however. The formation of a specific subfraction of HDL, termed preβ-HDL, appeared to be increased in plasma from transgenic mice. Although preβ-HDL is a quantitatively minor HDL subfraction, it is believed to be a very efficient acceptor of cellular cholesterol and therefore an elevated production of preβ-HDL might result in a strong induction of reverse cholesterol transport [15]. Therefore, we suggested that PLTP increased the anti-atherogenic potential of HDL [14]. Subsequently Jiang and co-workers showed that PLTP deficiency was anti-atherogenic, however [16], in spite of decreased levels of plasma HDL. The effect was attributed to a reduced production of apolipoprotein (apo)B containing lipoproteins by the liver. Indeed, we provided further evidence of an atherogenic role of PLTP in studies in a series of PLTP transgenic mouse lines with increasing levels of plasma PLTP activity, showing a PLTP-dose dependent enhancement of atherosclerosis [17]. We also provided in vivo evidence that PLTP is involved in the secretion of very low density lipoproteins (VLDL) [17], [18], but there was no PLTP-dose dependent increase of this effect in the series of PLTP transgenic mice tested [17]. In contrast, we observed a clear PLTP-dose dependent reduction of plasma HDL levels in parallel to the induction of atherosclerosis and therefore we concluded that elevated plasma PLTP activity in transgenic mice is atherogenic because it decreases plasma HDL. PLTP could also influence the atherogenicity of plasma lipoproteins by decreasing the vitamin E content of apoB-containing lipoproteins, resulting in increased susceptibility for oxidation [19].

PLTP is expressed in a wide variety of cells and tissues in humans [20], mice [21] and human PLTP transgenic mice [14]. In addition, the presence of PLTP in human atherosclerotic lesions has been demonstrated [22], [23], which probably originates from macrophages. In order to elucidate the role of PLTP derived from bone marrow derived cells, including macrophages, to atherosclerosis, we performed in vitro studies with macrophages from PLTP transgenic mice and performed bone marrow transplantations from PLTP transgenic mice to low density lipoprotein (LDL) receptor deficient mice and subsequently studied the process of diet-induced atherosclerosis as influenced by these bone marrow transplantations.

## Methods

### Mice

LDLR^−/−^ mice were obtained from the Jackson Laboratory (Bar Arbor, ME) and were in C57BL6/J background. Mice expressing enhanced green fluorescent protein (EGFP) under the control of the chicken beta-actin promotor and cytomegalovirus enhancer were originally generated by Okabe et al. and were in C57BL6/J background [24], [25]. Human PLTP transgenic mice were generated in our laboratory as described before and are derived from the P4 line [17]. The transgene in these animals contains the complete human PLTP gene, including approximately 15 kb 5′ natural flanking region and 3 kb 3′ natural flanking region. The transgene is driven by its autologous human promoter. PLTP transgenic mice were crossbred to C57BL6/J background for >15 generations. Subsequently, homozygous PLTP transgenic mice were obtained by crossbreeding hemizygous PLTP transgenic mice. Animals were provided with food and water ad libitum. Food was either regular chow, or a high fat, high cholesterol diet containing 40% (w/w) sucrose, 15% (w/w) cocoa butter, and 0.25% (w/w) cholesterol (diet W; Hope Farms, Woerden, The Netherlands). The numbers of animals used for measurements are indicated in the Figure legends. Only male mice were used. All of the procedures in this study were in accordance with national and institutional guidelines.

### Bone marrow transplantation

On the day of donor cell injection, LDLR^−/−^ mice of 12 weeks old were conditioned by 900 rads of γ-irradiation from a ^137^Cs source, which was administered in a split dose, with a 3 hours interval. Cells were injected i.v. into the tail veins. Each recipient received 5 × 10^6^ bone marrow cells isolated from the femurs and tibias of the primary donors. Injected animals were provided with 0.16% Neomycin (Sigma-Aldrich)-supplemented water. Animals were kept on a regular chow diet until 9 weeks after transplantation. During the next 9 weeks, animals were fed a high fat, high cholesterol diet.

### Analysis of plasma lipids, lipoproteins, PLTP concentration and PLTP activity

Blood samples were obtained via orbital bleedings after an overnight fast at 1 week before the bone marrow transplantation, just before the start of the diet and at the end of the 9 weeks high fat, high cholesterol diet feeding. Plasma lipids (total cholesterol, phospholipids and triglycerides) were measured using commercially available kits (Wako Chemicals). Lipoprotein fractions were obtained by ultracentrifugation of plasma samples in a Beckman 42.2 Ti rotor (42000 rpm, 3 h, 12°C) at d = 1.063 g/ml. Tubes were sliced, and two fractions were collected: non-HDL (VLDL+LDL), d<1.063 g/ml; and HDL, d>1.063 g/ml. PLTP activity in plasma was measured as described previously [14] and expressed in arbitrary units (AU). 1 AU is equivalent to the activity found in human reference plasma, which is 14 µmol/ml/h. PLTP concentration was measured by ELISA as described [18], using PLTP antibodies which were kindly donated by Dr. H. Hattori (BML Incorporated, Saitama, Japan). PLTP specific activity was calculated as the ratio (PLTP activity in AU)/(human PLTP concentration in mg/L).

### Histological analyses and quantification of atherosclerotic lesions

Animals were anesthetized using isoflurane, and in situ fixation was performed via the left ventricle of the heart using phosphate-buffered formaldehyde (4%, v/v). Sectioning of the aortic root, hematoxilin/eosin staining, the collection of digital images and the quantification of atherosclerotic lesions were performed as described before [26].

Immunohistochemistry was performed using antibodies directed against CD68 to detect macrophages (AbD Serotec, Kidlington, UK), against CD31 to detect endothelial cells (Hycult Biotechnology BV, Uden, The Netherlands), or against vascular smooth muscle cells alpha-actin (Sigma Chemical Co., Zwijndrecht, The Netherlands) or against PLTP (kind gift of Dr. Matti Jauhiainen, Helsinki, Finland).

### Cell culture studies

For the isolation of primary macrophages mice were i.p. injected with 3 mL of 40.5 g/L Bacto® Brewer Thioglycollate Medium (Difco International B.V., Leeuwarden, The Netherlands). After three days the animals are anesthetized using isoflurane and macrophages are isolated from the peritoneal cavity in 0.34 M sucrose. Cells were cultured in Dulbecco's Modified Eagle Medium (DMEM, Gibco, Invitrogen, Breda, The Netherlands) supplemented with 10% (v/v) fetal bovine serum and 100 U/mL penicillin/streptomycin. Cells were seeded on 6 wells plates (3×10^6^ cells/well) and kept in culture for 24 hours. Alternatively, macrophages derived from bone marrow in vitro were obtained by isolating bone marrow cells from tibias and femurs, seeding the cells on 6-wells plates (3×10^5^ cells/well) and culturing them in the presence of 10 ng/ml Macrophage Colony Stimulating Factor (Biosource International, Camarillo CA, USA) [27]. The concentrations of TNF-α, IFN-γ, IL-4, or IL-10 in the supernatants were measured using commercially available ELISAs (BD Biosciences Pharmingen, Alphen aan den Rijn, The Netherlands). PLTP activity was measured after 24 hours of culture in the supernatant with the same assay as used for the PLTP activity measurements in plasma. In some cases, cells were challenged by adding 100 ng/mL bacterial lipopolysaccharide (from E. coli serotype 0111:B4, Sigma, Chemical Co., Zwijndrecht, The Netherlands). In order to measure the cholesterol loading and efflux capacity [28], macrophages were seeded on 3 cm dishes (3×10^6^ cells/dish). After 2 hours, the medium was replaced with DMEM supplemented with 1% (v/v) fetal bovine serum, 100 U/mL penicillin/streptomycin, 30 µg/mL acetylated LDL (acLDL) [29], 1 µg/mL Acyl CoA:cholesterol acyltransferase inhibitor (gift from Sandoz AG, Basel, Switzerland) and 0.33 µCi [^3^H]-cholesterol/mL for 22 hours. Efflux was monitored by incubating these loaded cells in DMEM containing 0.2% (w/v) fat-free BSA (Sigma Chemical Co., #6003, Zwijndrecht, The Netherlands) and 100 µg/mL human HDL and sampling the medium at 1, 2, 4, and 6 hours. At the end of the efflux study, cell associated radioactive cholesterol was measured and the percentage of cholesterol efflux was calculated. Cells were lysed in 0.1 M NaOH/ 0.5% (w/v) SDS and cellular protein was determined using the Lowry method [30].

### Statistics

All of values are expressed as mean±S.E. Statistical analyses are by one-way analysis of variance with Bonferroni multiple comparison tests performed with Intercooled Stata 6.0 software (Stata Corp., College Station, TX, USA).

## Results

### PLTP production by mouse macrophages

**Figure 1:**
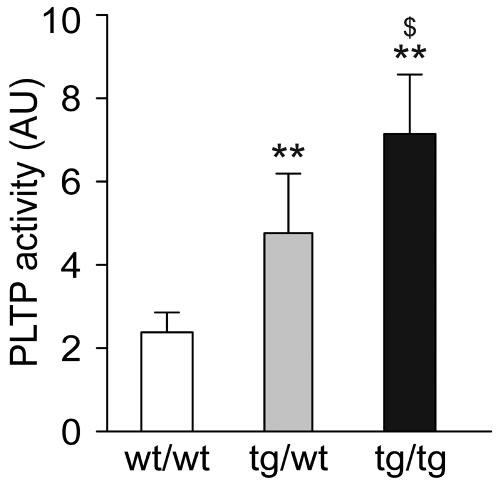
PLTP production by mouse macrophages. Peritoneal macrophages were collected from either C57BL/6J wild type (wt/wt), hemizygous huPLTP transgenic (tg/wt) and homozygous huPLTP transgenic (tg/tg) mice and kept in culture for 24 hours. Subsequently, PLTP activity was measured in a sample of the culture medium. PLTP activity is expressed as arbitrary units (AU; 1 AU is equivalent to 14 µmol/mL/h). N = cells from 6 mice per group. ** *p*<0.005 versus wt/wt; $ *p*<0.01 versus tg/wt.

In a first set of experiments, peritoneal macrophages were collected from three groups of mice: wild type control animals (PLTP^wt/wt^), hemizygous human PLTP transgenic mice (huPLTP^tg/wt^) and homozygous human PLTP transgenic mice (huPLTP^tg/tg^). Macrophages were kept in culture for 24 hours before PLTP activity was measured in the culture medium. As shown in Fig. 1, PLTP activity could be measured in the medium of cells from PLTP^wt/wt^ mice, demonstrating that PLTP is produced and excreted by macrophages. When macrophages from huPLTP^tg/wt^ and huPLTP^tg/tg^ mice were tested, increased PLTP activity was found (+125% and +233%, respectively, both *p*<0.01). From these results we conclude that macrophages from transgenic PLTP mice have an increased production and secretion of the transgenic protein.

### Effects of bone marrow transplantation on plasma PLTP activity

In order to study the contribution of PLTP expression by bone marrow derived cells on plasma PLTP activity, we performed bone marrow transplantation experiments from PLTP^wt/wt^, huPLTP^tg/wt^ and huPLTP^tg/tg^ mice to lethally irradiated LDL-receptor deficient (LDLR^−/−^) mice. Following the transplantation, animals were allowed to recover for 9 weeks. Subsequently, animals were fed a high fat, high cholesterol diet for another 9 weeks period. Blood samples were collected one week before irradiation, just before starting the diet and at the end of the 9 weeks diet period (time points -10 weeks, 0 weeks and 9 weeks, relative to the start of the diet, respectively). In addition to the transplanted animals, groups of LDLR^−/−^ mice and LDLR^−/−^/huPLTP^tg/wt^ mice were subjected to the same series of experiments, except the irradiation and bone marrow transplantation. Blood samples were collected at 0 weeks and 9 weeks. Animals were age matched to the mice in the transplantation groups.

**Figure 2:**
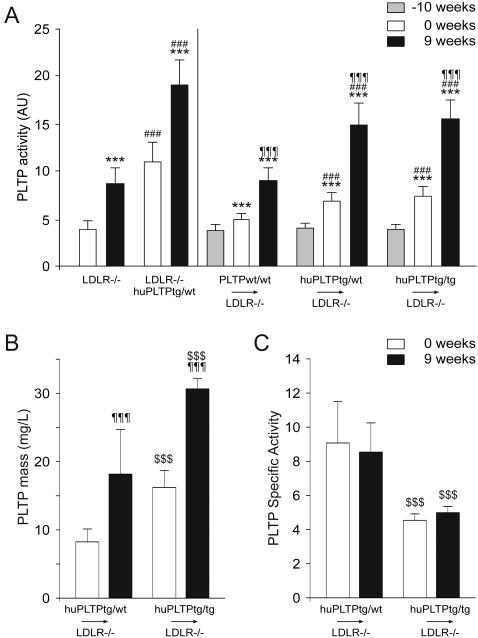
Plasma PLTP levels. A. PLTP activity was measured in plasma samples and expressed in arbitrary units as described in the legend of Fig. 1. Left panel: plasma samples were collected from non-transplanted LDLR^−/−^ and LDLR^−/−^/huPLTP^tg/wt^ mice just before the start of the high fat, high cholesterol diet (0 weeks, white bars) and after 9 weeks of the diet (9 weeks, black bars). *** *p*<0.001 versus 0 weeks (same genotype), ### *p*<0.001 versus LDLR^−/−^ mice (on the same diet). Right panel: plasma samples were collected from PLTP^wt/wt^→LDLR^−/−^, huPLTP^tg/wt^→LDLR^−/−^, and huPLTP^tg/tg^→LDLR^−/−^ recipient mice at 1 week before the start of the transplantation procedure (i.e. at 10 weeks before the start of the diet: −10 weeks, grey bars), just before the start of the diet (0 weeks, white bars) and after 9 weeks of diet (9 weeks, black bars). *** *p*<0.001 versus −10 weeks (same genotype); ### *p*<0.001 versus PLTP^wt/wt^→LDLR^−/−^ mice (on the same diet); ¶¶¶ *p*<0.001 versus 0 weeks (same genotype). B. Mass of human PLTP in plasma from mice was measured by ELISA as described in Methods in the Supplemental Data. C. Specific Activities of PLTP were calculated using the ratio between the activity in AU and the mass in mg/L. ¶¶¶ *p*<0.001 versus 0 weeks (same genotype); $$$ *p*<0.001 versus PLTP^tg/wt^→LDLR^−/−^ mice (on the same diet). N = 11–15 mice per group.

As shown in Fig. 2A (left panel), plasma PLTP activity in LDLR^−/−^/huPLTP^tg/wt^ mice were approximately 3-fold higher than in LDLR^−/−^ mice when fed a chow diet (0 weeks). After feeding the animals a high fat, high cholesterol diet for 9 weeks, plasma PLTP activity in both LDLR^−/−^ and LDLR^−/−^/huPLTP^tg/wt^ mice was increased by about 2-fold when compared to chow fed animals of the same genotype.

Fig. 2A (right panel) also shows the plasma PLTP activity in transplanted animals. The transplantation procedure itself resulted in a significant increase in PLTP activity, ranging from 29% in the PLTP^wt/wt^→LDLR^−/−^ mice to 88% in the huPLTP^tg/tg^→LDLR^−/−^ mice (all *p<*0.001). After feeding the animals a high fat, high cholesterol diet, a further increase in plasma PLTP activity was observed ranging from 82% in the PLTP^wt/wt^→LDLR^−/−^ mice to 113% in the huPLTP^tg/tg^→LDLR^−/−^ mice (all *p<*0.001). Under both dietary conditions, plasma PLTP activity did not differ between huPLTP^tg/wt^→LDLR^−/−^ and huPLTP^tg/tg^→LDLR^−/−^ mice.

Fig. 2B shows the concentration of human PLTP in plasma from transplanted animals. As there is no ELISA available for mouse PLTP, mass of plasma PLTP could only be measured in the huPLTP^tg/wt^→LDLR^−/−^ and huPLTP^tg/tg^→LDLR^−/−^ mice. The PLTP concentration appeared to be approximately twice as much in the latter animals. The high fat, high cholesterol diet resulted in a two-fold increase in PLTP mass. The specific activity of plasma PLTP was not affected by the diet (Fig. 2C). Interestingly however, the specific activity in the huPLTP^tg/tg^→LDLR^−/−^ mice was significantly lower than in the huPLTP^tg/wt^→LDLR^−/−^ mice, implying that part of the huPLTP in the huPLTP^tg/tg^→LDLR^−/−^ mice is in the inactive form.

### Effects of bone marrow transplantation on plasma lipids and lipoproteins

**Figure 3:**
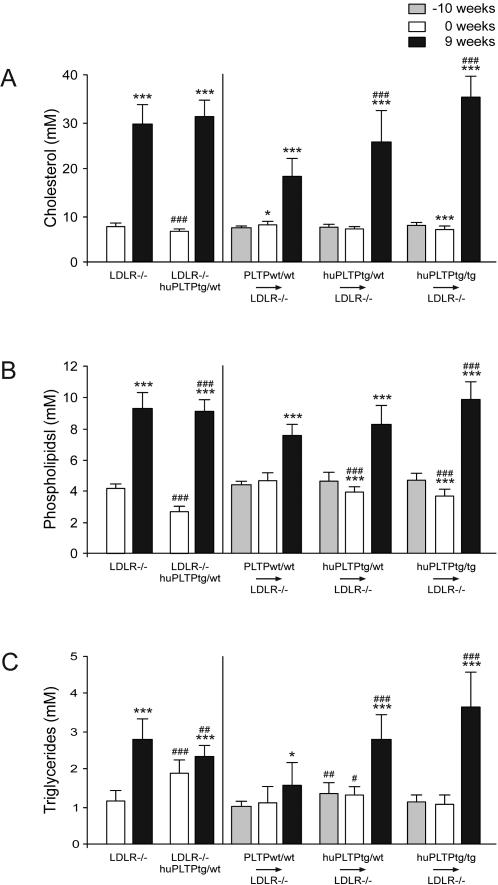
Plasma lipid levels. Plasma levels of cholesterol (A), phospholipids (B) and triglycerides (C) were measured as described in Methods. Left panels: Plasma samples were collected from non-transplanted LDLR^−/−^ and LDLR^−/−^/huPLTP^tg/wt^ mice just before the start of the high fat, high cholesterol diet (0 weeks, white bars) and after 9 weeks of the diet (9 weeks, black bars). *** *p*<0.001 versus 0 weeks (same genotype), ### *p*<0.001 versus LDLR^−/−^ mice (on the same diet). Right panels: plasma samples were collected from PLTP^wt/wt^→LDLR^−/−^, huPLTP^tg/wt^→LDLR^−/−^, and huPLTP^tg/tg^→LDLR^−/−^ recipient mice at 1 week before the start of the transplantation procedure (i.e. at 10 weeks before the start of the diet: −10 weeks, grey bars), just before the start of the diet (0 weeks, white bars) and after 9 weeks of diet (9 weeks, black bars). N = 11–15 mice per group. * *p*<0.05, *** *p*<0.001 versus −10 weeks (same genotype); ### *p*<0.001 versus PLTP^wt/wt^→LDLR^−/−^ mice (on the same diet).

**Figure 4:**
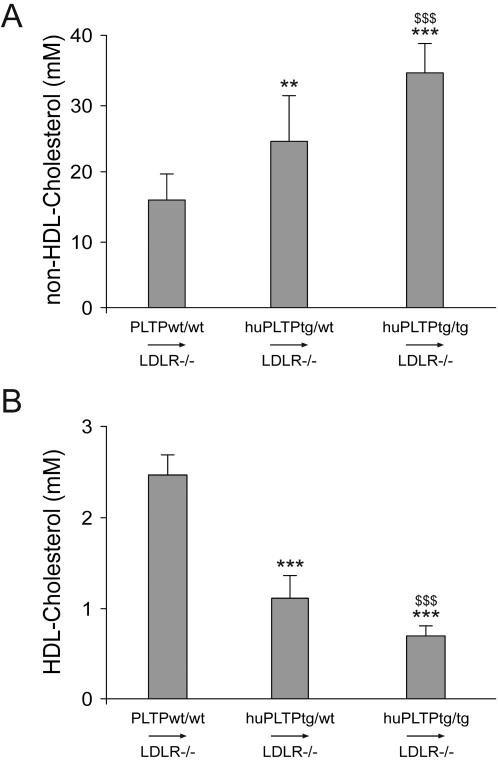
Plasma levels of non-HDL cholesterol and HDL cholesterol. Plasma was collected from PLTP^wt/wt^→LDLR^−/−^, huPLTP^tg/wt^→LDLR^−/−^, and huPLTP^tg/tg^→LDLR^−/−^ recipient mice at the end of the high fat, high cholesterol diet period (i.e., at time of sacrifice) and separated into two fractions: d<1.063 g/L (non-HDL) and d>1.063 g/L (HDL) by ultracentrifugation. In both fractions, the cholesterol concentration was measured. A: non-HDL cholesterol; B: HDL-cholesterol. N = 11–15 mice per group. ** *p*<0.01, *** *p*<0.001 versus PLTP^wt/wt^→LDLR^−/−^ mice; $$$ *p*<0.001 versus huPLTP^tg/wt^→LDLR^−/−^ mice.

When fed a regular chow diet, plasma cholesterol levels were slightly decreased in LDLR^−/−^/huPLTP^tg/wt^ mice when compared to LDLR^−/−^ mice (Fig. 3A, left panel). However, this difference was absent after feeding the animals a high fat, high cholesterol diet for 9 weeks. In the transplanted animals, no major differences in plasma cholesterol concentration were observed between the various groups on chow diet (Fig. 3A, right panel). However, after feeding the animals a high fat, high cholesterol diet for 9 weeks an increase in cholesterol concentration was observed in PLTP^wt/wt^→LDLR^−/−^, huPLTP^tg/wt^→LDLR^−/−^, and huPLTP^tg/tg^→LDLR^−/−^ mice. Effects on plasma phospholipids and triglycerides largely mirrored those on plasma cholesterol (Fig. 3B,C). In order to further investigate these differences, HDL and non-HDL was separated in plasma samples of the animals after 9 weeks on a high fat, high cholesterol diet. The mice transplanted with huPLTP transgenic bone marrow cells showed an increase in non-HDL cholesterol (Fig. 4A) and a decrease in HDL-cholesterol (Fig. 4B) when compared with the mice transplanted with wild type bone marrow cells.

### Effects of bone marrow transplantation on atherosclerosis

**Figure 5:**
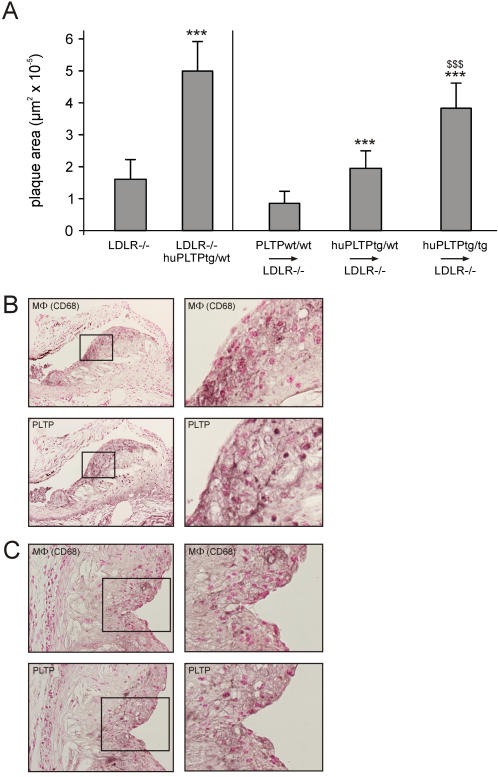
Atherosclerosis in non-transplanted and transplanted mice. A. Plaque area was measured in sections from the aortic root (see Methods). Left panel: Plaque area in non-transplanted LDLR^−/−^ and LDLR^−/−^/huPLTP^tg/wt^ mice. *** *p*<0.001 versus LDLR^−/−^ mice. Right panel: Plaque area in PLTPwt/wt→LDLR^−/−^, huPLTP^tg/wt^→LDLR^−/−^, and huPLTP^tg/tg^→LDLR^−/−^ recipient mice. N = 11–15 mice per group. *** *p*<0.001 versus PLTP^wt/wt^→LDLR^−/−^ mice; $$$ *** *p*<0.001 versus huPLTP^tg/wt^→LDLR^−/−^ mice. B, C. Immunohistochemistry of atherosclerotic lesions from non-transplanted LDLR^−/−^/huPLTP^tg/wt^ mice (B) and from transplanted huPLTP^tg/tg^→LDLR^−/−^ mice (C), stained for macrophages (MΦ) with anti-CD68 and for PLTP and counterstained with Nuclear-fast red (serial sections). The right panels are magnifications of the boxed parts in the left panels. Original magnifications: 100x (B) and 250x (C).

After feeding the animals a high fat, high cholesterol diet for 9 weeks, atherosclerosis was quantified by measuring the area of atherosclerotic lesions in the aortic root. In agreement with previous studies from our laboratory [17], [31], we found that elevated levels of PLTP result in a strong increase (312%) in diet-induced atherosclerosis in non-transplanted mice (Fig. 5A, left panel; *p<*0.001). In transplanted mice, a clear increase in atherosclerotic lesion area was observed (Fig. 5A, right panel; all differences *p<*0.001). Compared to PLTP^wt/wt^→LDLR^−/−^ mice, huPLTP^tg/wt^→LDLR^−/−^ mice have an increase of 128% in atherosclerotic lesion area, while huPLTP^tg/tg^→LDLR^−/−^ mice have an increase of 348% in atherosclerotic lesion area.

The presence of PLTP expressing macrophages in the aortic lesions was confirmed by immunohistochemistry. As illustrated in Fig. 5, the plaques from both non-transplanted LDLR^−/−^/huPLTP^tg/wt^ mice (Fig. 5B) as those from recipient huPLTP^tg/tg^→LDLR^−/−^ mice (Fig. 5C) contain considerable amounts of macrophages while PLTP is co-localized in similar areas of the lesions.

**Figure 6:**
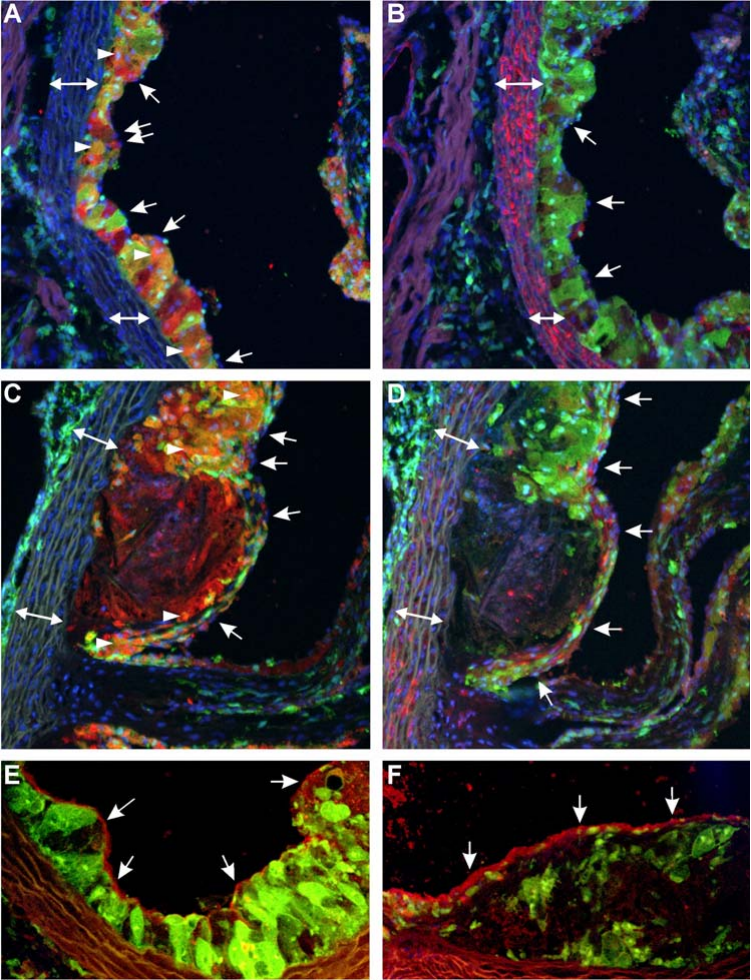
Transplantations with β-actin GFP mice→LDLR^−/−^ mice. A–D: Early lesions (A,B) and advanced lesions (C,D) with donor cells expressing GFP (green), CD 68 (marker for macrophages) in red (A,C) and α-actin (marker for VSMCs) in red (B,D) and nuclei stained with DAPI (blue). Endothelial cells covering the lesions do not express GFP (nuclei indicated with arrows). Co-localization of GFP and CD68 results in an orange color (arrowheads). The necrotic core in the advanced lesion (located centrally in C and D) is diffusely positive for CD68 but does not show any GFP signal. The media is marked with a double arrow (↔). E,F: Early lesion (E) and advanced lesion (F) with donor cells expressing GFP (green), and CD 31 (marker for endothelial cells) in red (arrows). Representative pictures from N = 6 animals are shown. Original magnifications: 200X.

In order to be more conclusive on the nature of the bone marrow derived cells, we performed an additional set of experiments using actin-EGFP mice as donors and LDLR^−/−^ mice as acceptors, using exactly the same experimental procedure to obtain diet-induced atherosclerosis. Sections from the aortic root were inspected using confocal microscopy after immunohistochemistry with antibodies directed against CD68 as a marker for macrophages or VSMC α-actin as a marker for VSMCs. DAPI staining was used to visualize nuclei. Bone marrow derived donor cells, identified by the GFP signal, are clearly present in the subendothelial space of the early lesions and co-localize to a great extent with CD68 (Fig. 6A,B). In the advanced lesions, the cell-rich regions show significant co-localization of the GFP and CD68 signals, while the necrotic core stills allows detection of CD68, but GFP is no longer expressed (Fig. 6C,D). In early lesions, VSMCs are almost exclusively located in the media (Fig. 6B). In advanced lesions, VSMCs can be detected in the fibrous cap (Fig. 6D), but they do not co-localize with GFP, demonstrating that also the VSMCs in the lesions are not donor-derived. As shown in Fig. 6A–D, isolated nuclei can be detected of endothelial cells covering the lesions without GFP signal, demonstrating that these cells are not donor-derived. Positive staining for endothelial cells was performed by using an antibody directed against CD31 (Fig. 6E,F). The endothelial lining in early (Fig. 6E) and in advanced (Fig. 6F) lesions is continuous and does not express GFP, implying that the endothelial cells are not donor derived.

### Characterization of PLTP expressing macrophages

In order to investigate the mechanism by which mice transplanted with bone marrow cells expressing elevated levels of PLTP develop more atherosclerosis, we performed a series of in vitro studies using primary macrophages from PLTP^wt/wt^, huPLTP^tg/wt^, and huPLTP^tg/tg^ mice, respectively.

**Figure 7:**
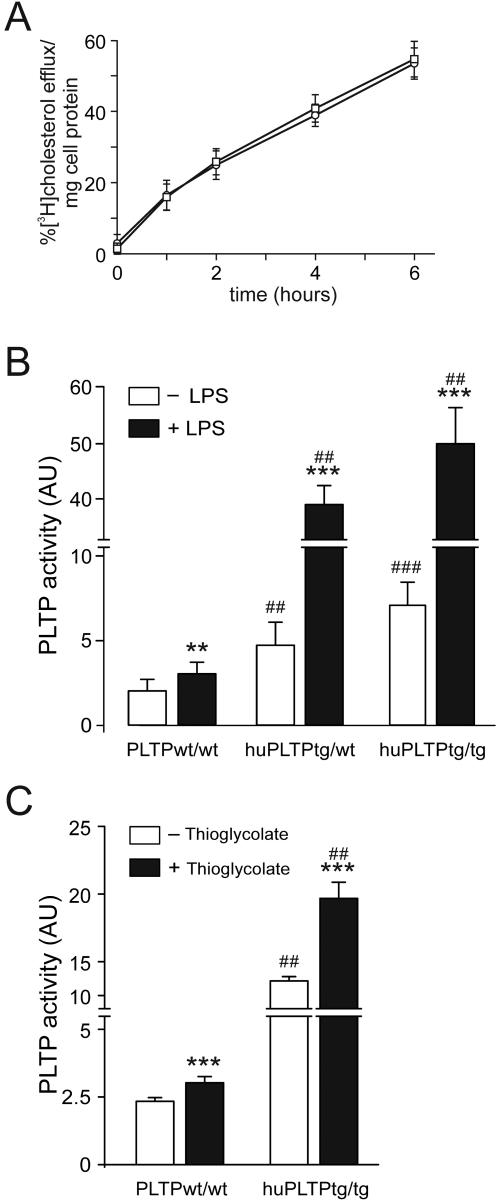
Characteristics of mouse macrophages. A. Cholesterol efflux from cultured peritoneal macrophages. Cells were loaded with radioactive cholesterol as described in Methods. Subsequently, culture medium with human HDL as an acceptor was added. Left panel: Radioactivity was measured in aliquots from the cultured medium taken at 0, 1, 2, 4, and 6 hours. Circles: macrophages from C57BL/6J mice, squares: macrophages from huPLTP^tg/tg^ mice. Differences were not statistically significant. B. PLTP activity measured in the medium of macrophages cultured for 24 hours in the absence (white bars) or presence (black bars) of 100 ng/mL LPS. Cells from 6 mice were used per group. C. PLTP activity measured in plasma samples from mice treated with thioglycolate. N = 6 mice per group. ** *p*<0.005, *** *p*<0.001 versus cells without LPS or thioglycolate (same genotype), ## *p*<0.005, ### *p*<0.001 versus PLPT^wt/wt^ (same culture medium, with or without LPS or thioglycolate).

First we examined whether PLTP expression in macrophages affects their capacity to perform cellular cholesterol efflux. To this end, macrophages were loaded with radiolabeled cholesterol using acLDL and efflux to the medium was studied during six hours using human HDL as an acceptor. There was no difference in cholesterol loading between macrophages from PLTP^wt/wt^ and huPLTP^tg/tg^ mice (42.87+/−8.25 versus 44.26+/−8.67% [^3^H]cholesterol/mg cell protein). No difference in efflux to HDL was observed (Fig. 7A). Consequently, the amount of radioactive cholesterol that remained cell-associated at the end of the efflux period was also similar (not shown).

Possible differences between the inflammatory status of the macrophages were studied by comparing the level of the cytokines, TNF-α, IFN-γ, IL-4, and IL-10, by ELISA. No differences were found in medium from macrophages obtained from PLTP^wt/wt^, huPLTP^tg/wt^, and huPLTP^tg/tg^ mice before or after stimulation with LPS (results not shown). These results indicate that there is no difference in cytokine production by macrophages from these mice.

Subsequently, we studied whether activated macrophages have different PLTP activity. Therefore, we challenged the macrophages with bacterial lipopolysaccharide (LPS), and measured PLTP activity in the medium from the stimulated macrophages. As shown in Fig. 7B, stimulation of macrophages from PLTP^wt/wt^ mice with LPS resulted in approximately 70% increase in PLTP activity. Unstimulated macrophages from huPLTP^tg/wt^ or huPLTP^tg/tg^ mice secreted approximately 2.5 to 4 fold more PLTP in the culture medium than macrophages from PLTP^wt/wt^ mice. LPS stimulation resulted in a further 7.5 fold increase of PLTP activity in the culture medium from macrophages from huPLTP^tg/wt^ or huPLTP^tg/tg^ mice. In order to investigate whether this in vitro effect would be reflected by similar effects in vivo, we measured plasma PLTP activity levels in both PLTP^wt/wt^ and huPLTP^tg/tg^ mice that were treated with thioglycolate. In both cases, this treatment resulted in a statistically significant (P<0.002) increase in plasma PLTP activity (Fig. 7C).

Finally, we cultured macrophages from bone marrow in order to consolidate these findings. Comparing macrophages from PLTP^wt/wt^ and huPLTP^tg/tg^ mice, we found much more PLTP activity in the medium of the huPLTP^tg/tg^ cells. PLTP activity could be significantly increased in cells from both PLTP^wt/wt^ and huPLTP^tg/tg^ mice after treatment with LPS (not shown).

## Discussion

The main findings of this study are: (1) Mouse macrophages express and secrete PLTP while mouse macrophages from human PLTP transgenic mice express and secrete increased levels of PLTP; (2) Bone marrow transplantations using PLTP transgenic mice as donors induce elevated plasma PLTP activity levels in the acceptor mice; (3) PLTP overexpression results in decreased plasma levels of HDL cholesterol and in increased plasma levels of non-HDL cholesterol; (4) Transplantation with PLTP expressing bone marrow cells results in increased (2–4.5 fold) atherosclerosis with abundant intra-plaque presence of PLTP.

The potential relevance of PLTP production by macrophages is emphasized by three independent studies, showing that PLTP protein is present in human atherosclerotic lesions [22], [23], [32]. Although intriguing, the presence of PLTP in atherosclerotic lesions per se does not prove a direct involvement in the process of atherosclerosis and therefore the significance of these findings is unclear. In humans, the causal relation between PLTP and atherosclerosis is still uncertain [33]. Plasma PLTP activity is elevated in patients suffering from diabetes mellitus type 1 [34] and type 2 [35], [36]. In addition, increased PLTP is found in obese individuals [37]–[40] while it is decreased with weight loss [41]. The elevated plasma PLTP activity in patients with type 2 diabetes mellitus is positively correlated with the carotid intima-media thickness [42].

In order to find out what the contribution of PLTP expressing macrophages to diet induced atherosclerosis would be, we performed bone marrow transplantation experiments.

9 weeks after bone marrow transplantation, a moderate increase in plasma PLTP activity was found in the animals transplanted with PLTP^wt/wt^ cells. In the animals transplanted with PLTP transgenic cells, a further increase was observed. Feeding the animals a high fat, high cholesterol diet for 9 weeks, resulted in a striking increase in plasma PLTP activity. It has been demonstrated that cholesterol loading of macrophages induces a strong (2–3 fold) induction of PLTP mRNA, PLTP protein secretion and an increased PLTP activity in the medium conditioned by these cells [23], [32]. Our results show that in animals fed a high fat, high cholesterol diet, plasma from huPLTP^tg/wt^→LDLR^−/−^ and huPLTP^tg/tg^→LDLR^−/−^ mice have a 66–75% increase in PLTP activity when compared to plasma from PLTP^wt/wt^→LDLR^−/−^ mice. Thus we conclude that a considerable amount of macrophage PLTP is excreted into the blood.

An increase in plasma PLTP activity affects plasma lipids. Plasma cholesterol is not affected by the moderate increase in PLTP activity caused by the bone marrow transplantation procedure itself. After 9 weeks of high fat, high cholesterol diet plasma cholesterol is elevated, as expected, but a further increase is observed in the huPLTP^tg/wt^→LDLR^−/−^ and huPLTP^tg/tg^→LDLR^−/−^ mice. So, PLTP expression by bone marrow derived cells affects not only plasma PLTP activity levels, but also plasma lipids.

Because PLTP activity can be directly measured in plasma, our experiments clearly show that PLTP is excreted by bone marrow derived cells into the plasma compartment. Probably this is the cause of the observed decrease in plasma HDL cholesterol, which is in agreement with previous results from our laboratory [14], [17], [31] and others [43], [44], and which is probably caused by an enhanced catabolism of HDL [43]. Most likely, this effect will contribute to the observed enhanced atherosclerosis in mice transplanted with huPLTP transgenic bone marrow. In addition however, there might be a local effect of PLTP produced by macrophages in the vascular wall. Although PLTP overexpressing macrophages do not show any difference in cholesterol efflux capacity or cytokine production, a very strong induction of PLTP production is observed in macrophages treated with LPS. So, activated macrophages present in the inflammatory environment of atherosclerotic plaques probably excrete large amounts of PLTP. This is confirmed by immunohistochemistry data in the present study. The high local PLTP production might result in a further increase of the inflammatory status of the lesions. Additional support for this idea is provided by studies showing that PLTP deficiency is associated with anti-inflammatory effects [45], [46], and by a study in which an association was found between PLTP activity and inflammatory markers in patients with cardiovascular disease [47]. Alternatively, extracellular PLTP might act as a trap for HDL. PLTP is able to bind to the extracellular matrix proteoglycan biglycan [23] and thus act as a bridging molecule to bind HDL. In vitro studies showed that this action is independent of PLTP's phospholipid transfer activity. By trapping HDL in the atherosclerotic lesion, PLTP could interfere with the reverse cholesterol transport activity of HDL and thus enhance lipid accumulation, or actually, inhibit lipid removal, and hence stimulate the development of the plaque. It is uncertain whether this HDL retention may contribute to the decreased plasma levels of HDL observed in mice transplanted with huPLTP transgenic bone marrow.

While macrophage expression of PLTP has both systemic and local effects, both of which might be atherogenic, the steep increase in atherosclerotic lesion size of almost 2-fold between huPLTP^tg/wt^→LDLR^−/−^ and huPLTP^tg/tg^→LDLR^−/−^ mice is not completely reflected by the differences in the systemic effects, as these mice have similar plasma PLTP activities. This suggests that local effects of PLTP expression might indeed play an important role. In addition, the huPLTP^tg/tg^→LDLR^−/−^ mice show a reduced specific activity of PLTP. This could indicate that the huPLTP^tg/tg^→LDLR^−/−^ mice have relatively more inactive PLTP [48], which may not have phospholipids transfer activity, but which might still have some of the other effects described above. Also, PLTP secreted by the huPLTP^tg/tg^ bone marrow derived cells in the transplanted animals finds itself in a plasma environment with very little HDL. As active PLTP is normally bound to HDL, this situation could destabilize PLTP resulting in partial PLTP inactivation and in a decrease in PLTP specific activity.

It should be noted that bone marrow derived cells include cells other than macrophages, like endothelial cells and VSMCs. Therefore, we performed a set of control experiments with β-actin GFP donor mice. It became clear that using our experimental conditions, the contribution from bone marrow derived endothelial cells or VSMCs to the atherosclerotic lesion is undetectable, while there is a significant number of bone marrow derived macrophages in the lesions.

Recently, the susceptibility to diet-induced atherosclerosis was studied in mice after bone marrow transplantations with bone marrow cells from wild type and PLTP deficient mice. Strikingly different results were reported by two independent research groups, leading to opposite conclusions [49], [50]. While Valenta et al [49] propose an athero-protective role for macrophage derived PLTP, Vikstedt et al [50] show evidence for an atherogenic function of macrophage derived PLTP. Both groups used LDLR^−/−^ mice as acceptors, and either wild type or PLTP^−/−^ mice as donors. All mice had the same genetic background (C57BL/6J), and the same strain of PLTP deficient mice were used. When comparing mice transplanted with PLTP^−/−^ bone marrow with mice transplanted with wild type bone marrow, Valenta et al found an increase in atherosclerotic lesion area in of 30% (measured in the aortic valves) or 28% (measured in the entire aorta), while Vikstedt et al found a decrease of 29% (measured in the aortic valves). In both studies, plasma PLTP activity is lower in mice transplanted with PLTP^−/−^ bone marrow than in mice transplanted with wild type bone marrow when fed a chow diet, and the difference becomes larger in mice fed an atherogenic diet. In the study by Vikstedt et al this leads to a significant decrease in plasma cholesterol levels and an increase in HDL, which may explain the decreased atherosclerosis found in mice transplanted with PLTP^−/−^ bone marrow. In the study by Valenta et al there is increased atherosclerosis while plasma cholesterol levels are hardly changed. Thus, in the latter study postulated that plasma PLTP is atherogenic, while PLTP which is locally active in the vascular wall may have anti-atherogenic potential, possibly by local stimulation of macrophage cholesterol efflux.

Vikstedt et al have summarized the major differences in experimental set-up: 1) Vikstedt et al used female mice as acceptors while Valenta et al used male mice; 2) Vikstedt et al fed the mice an atherogenic diet with 0.25% cholesterol for 9 weeks while the diet used by Valenta et al contained 1.25% cholesterol and was fed for 16 weeks; 3) Vikstedt et al allowed the mice to recover from the bone marrow transplantation procedure for 8 weeks before the atherogenic diet was supplied while Valenta et al applied a recovery time of only 4 weeks. Vikstedt et al do not indicate if any of these differences could be the deciding factor to explain the different outcomes of their studies.

Very recently, Valenta et al confirmed their previous findings in a study in which they used LDLR^−/−^/PLTP^−/−^ mice as acceptors [51]. Here, they applied the same experimental set-up as in their earlier work. In the discussion of this recent paper, the differences with the experimental set-up followed by Vikstedt et al are listed, but there are no conclusions about what might really cause the different outcomes.

We believe that the difference in gender between the recipients in the studies using PLTP deficient mice is not a likely explanation for the conflicting results. Although it is well-known that the susceptibility of female and male mice to atherosclerosis is different, the reason for this phenomenon is unknown, and, to the best of our knowledge, there are no reports where opposite outcomes of an intervention with respect to atherosclerosis susceptibility between males and females have been described. In fact, we have shown in a previous study that in animals with elevated PLTP activity, both males and females have more atherosclerosis [52].

The difference in diet-regime may be an essential factor. The diet used by Valenta et al contains five times as much cholesterol as the diet used by Vikstedt et al and was applied much longer. Therefore, this is a significantly more aggressive way of induction of atherosclerosis. It is conceivable that this has affected the outcome of the study.

The difference in recovery time may be an essential factor as well. Vikstedt et al allowed the animals to recover for eight weeks, compared to four weeks in the study of Valenta et al. Four weeks is really a very short time, in which the hematopoietic system has not yet been stably reconstituted. It is conceivable, that PLTP deficiency affects the repopulation of monocytes/macrophages. Therefore, the development of atherosclerotic lesions may be affected by differences in recovery time. Also, the reconstitution of resident macrophages in lung and liver, both important sites of PLTP expression, may not yet be stable after only four weeks of recovery [53].

We studied the effects of elevated PLTP expression using transgenic mouse models that have been developed in our own laboratory. The interpretation of our results is independent from the conflicting results that have been reported using PLTP^−/−^ mice. Our experimental set-up is comparable to the one used by Vikstedt et al, as we also fed the mice a diet containing 0.25% cholesterol for 9 weeks, and we used an even slightly longer recovery period (9 weeks). Their results are in line with our findings, as we report an increase of atherosclerosis in mice transplanted with PLTP overexpressing bone marrow, while they found decreased atherosclerosis in mice transplanted with PLTP^−/−^ bone marrow cells.

In conclusion, we showed that elevated macrophage expression of PLTP results in increased atherosclerosis. This is probably directly related to the effects on plasma lipoproteins caused by PLTP expression by bone marrow derived cells. In addition, local effects in the atherosclerotic lesion could contribute to this process. These results indicate that the presence of PLTP in human atherosclerotic lesions probably contributes to the pathologic process and that inhibition of PLTP accumulation in atherosclerotic lesions may be a valuable target for therapy.
